# Suspected Testicular Torsion With an Atypical Presentation

**DOI:** 10.7759/cureus.89517

**Published:** 2025-08-06

**Authors:** Ahmad A Alnasser, Abdulrahman K Dawagee, Mohammed M Al Mutairi, Tarek A Swellam

**Affiliations:** 1 Department of Urology, Dammam Medical Complex, Dammam, SAU

**Keywords:** atypical findings, atypical presentation, testicular exploration, testicular pain, torsion testis

## Abstract

Testicular torsion is a urological emergency that occurs when the spermatic cord twists, cutting off blood supply to the testicle, and it requires immediate surgical intervention to prevent irreversible damage. Although it typically presents with sudden, severe scrotal pain and swelling, atypical presentations can complicate diagnosis and delay treatment. We report the case of a 25-year-old male who presented to the emergency department with a sudden onset of left lower abdominal pain and left inguinal swelling. Despite the absence of testicular tenderness, scrotal ultrasound revealed reduced blood flow to the left testicle, raising suspicion for torsion. Emergency scrotal exploration confirmed a torsed spermatic cord, which was successfully detorsed, and the testicle was secured with orchidopexy. Postoperative recovery was uneventful, and the patient was discharged in good condition. This report highlights the importance of maintaining a high index of suspicion for testicular torsion, even when symptoms deviate from the typical presentation, as early clinical recognition and prompt surgical intervention are essential to optimize outcomes and preserve testicular function.

## Introduction

Testicular torsion is a urologic emergency with potentially devastating consequences, and a delay in its diagnosis and treatment can lead to permanent loss of the affected testicle. To prevent this serious complication, healthcare providers must differentiate testicular torsion from other causes of acute scrotal pain. Early and accurate diagnosis is essential to ensure timely intervention and preserve testicular function. Testicular torsion causes severe and sudden testicular pain and occurs when the spermatic cord, which supplies blood to the testicle, twists and compromises vascular flow [1]. The condition has a bimodal distribution, with two distinct age peaks: the first during the neonatal period, typically within the first year of life, and the second during adolescence, usually between the ages of 13 and 16 years [2]. Notably, research suggests that up to half of boys ultimately diagnosed with torsion may have experienced previous episodes of testicular pain [3].

While severe and sudden scrotal pain is the classic symptom of testicular torsion, atypical presentations can also occur. In some cases, patients initially experience lower abdominal or groin pain that migrates to the scrotum over a few hours, potentially delaying diagnosis. Because timely intervention is critical for testicular salvage, any male patient presenting with acute lower abdominal pain, particularly adolescents, should undergo a testicular examination to exclude torsion. A prompt and thorough clinical assessment can help identify this urologic emergency and prevent irreversible testicular damage [1]. This report highlights the need for healthcare providers to maintain a high level of vigilance for testicular torsion, even when symptoms deviate from the classic presentation, as diagnostic delays can significantly impact outcomes.

## Case presentation

A 25-year-old male with no significant medical or surgical history presented to our emergency department (ED) with a sudden onset of left lower abdominal pain and left inguinal swelling that had begun four hours before presentation. He was initially referred to the general surgery service with a presumed diagnosis of a left incarcerated inguinal hernia. The pain was described as sudden, colicky in nature, localized to the left inguinal area, scored 9/10 in severity, and neither radiating nor shifting. The patient denied associated symptoms such as nausea, vomiting, changes in bowel habits, fever, heavy lifting, or trauma. A CT scan was performed, revealing no definite abdominal wall defect to suggest a hernia but showing a left-sided non-communicating hydrocele with engorgement of the left spermatic cord. He was subsequently referred to the urology service with a diagnosis of left hydrocele. All of these misdiagnoses led to a delay of almost two more hours. 

**Figure 1:**
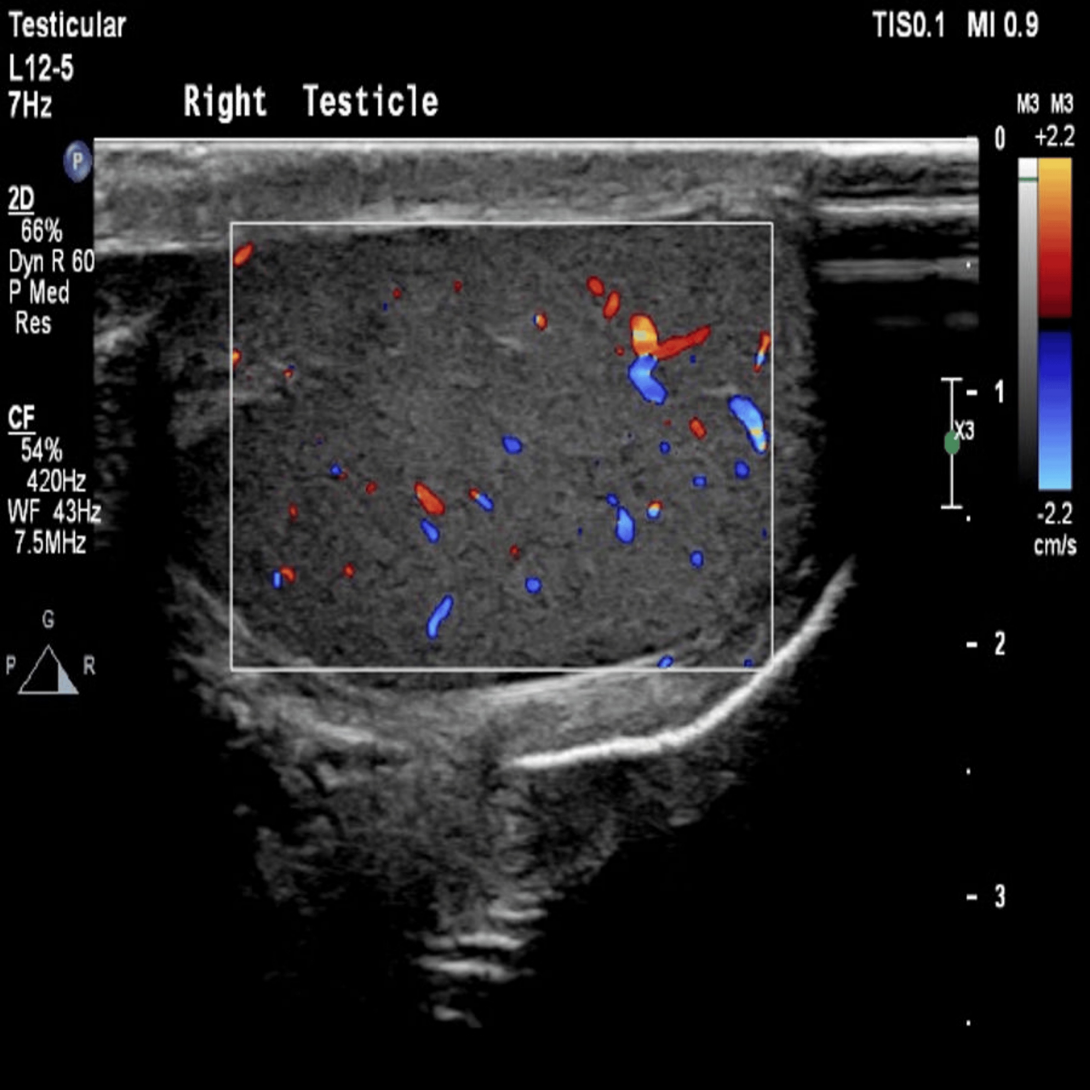
Ultrasound image of right testicle demonstrating homogeneous echogenicity with preserved vascularity

On examination, his vital signs were normal, and he was afebrile. Abdominal examination revealed a soft, non-distended abdomen with a tender, irreducible swelling measuring approximately 3 × 3 cm in the left groin region. The right testicle appeared normal; however, the left testicle was slightly elevated with a thickened spermatic cord palpable at its base, although without direct tenderness. Initial laboratory investigations revealed a white blood cell count of 10.8 × 10⁹/L, hemoglobin of 16.2 g/dL, and platelet count of 225 × 10⁹/L. Scrotal ultrasound demonstrated that the right testicle had preserved homogeneous echogenicity and vascularity, with no evidence of a hydrocele (Figure 1).

**Figure 2:**
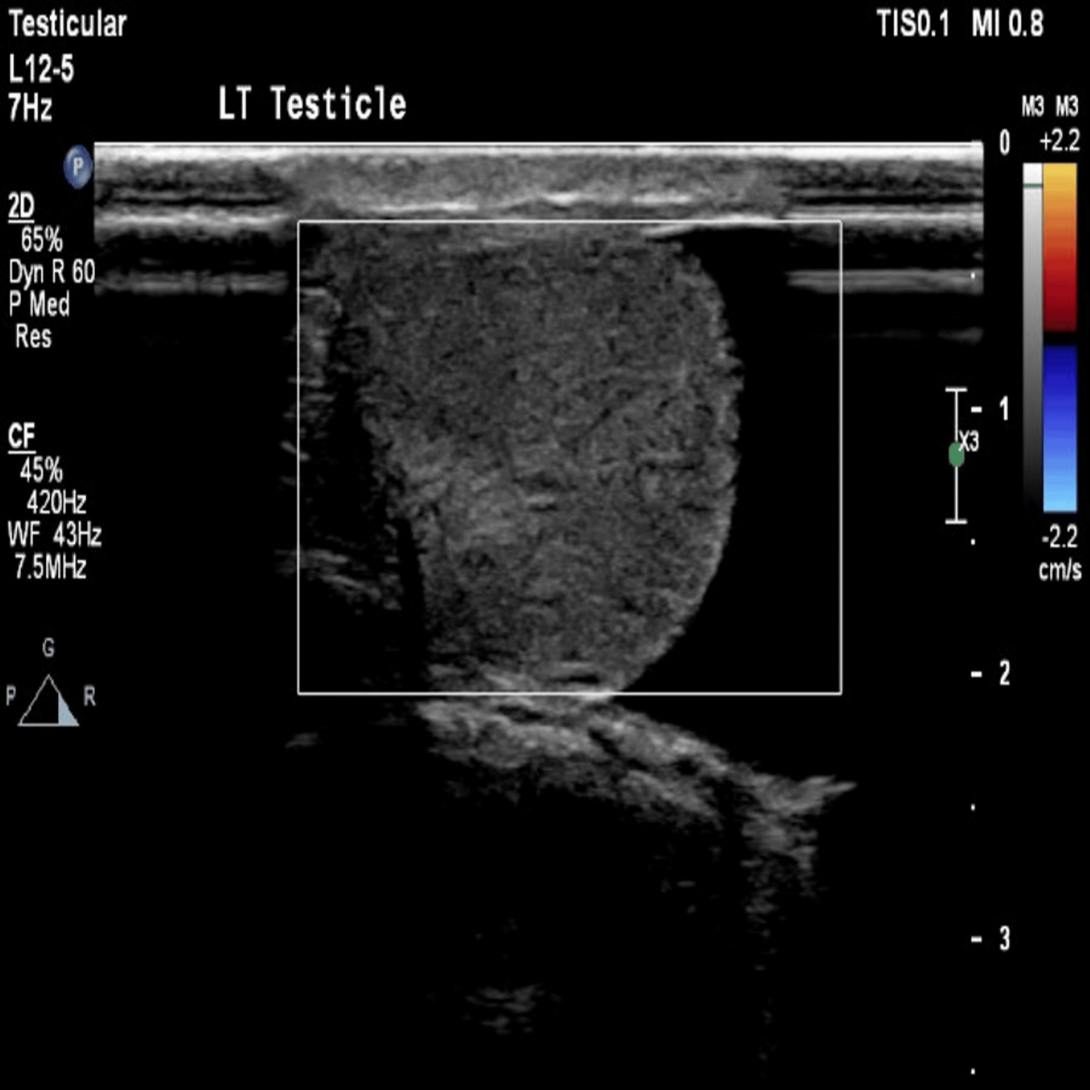
Ultrasound image of left testicle demonstrating hypoechoic relative to the right and lacks internal vascularity on color Doppler

In contrast, the left testicle appeared hypoechoic relative to the right, showed absent internal vascularity on color Doppler imaging, and was abnormally oriented. A left-sided hydrocele and a left epididymal tail cyst measuring 0.5 × 0.4 cm were also identified (Figure 2).

The patient underwent emergency scrotal exploration, which revealed torsion of the left spermatic cord and associated torsion of the testicular appendix. A long, twisted spermatic cord, suggestive of chronic retractile testis, was identified and detorsioned. The affected testicle was placed in a warm saline-soaked sponge, and high-flow oxygen therapy was administered to enhance perfusion. After 10-15 minutes, a notable improvement in testicular color was observed, indicating the restoration of blood flow. The torsed testicular appendix was excised, and a left orchidopexy was performed using non-absorbable sutures to prevent future torsion. Right orchidopexy was also performed through the same median raphe incision. The patient had an uneventful postoperative course and was discharged on the second postoperative day. At follow-up two weeks later, he reported doing well, experiencing only minimal scrotal discomfort during physical activity.

## Discussion

Testicular torsion is a urologic emergency that demands immediate attention to avoid the risk of testicular loss. The condition commonly presents with sudden, severe scrotal pain and swelling, but atypical presentations can complicate diagnosis and delay treatment. This report highlights the significance of recognizing both typical and atypical presentations, drawing on our case and insights from the study titled "Acute testicular torsion: A critical analysis of presentation, management and outcome in southeast Nigeria" [4]. The typical presentation of testicular torsion involves the sudden onset of severe scrotal pain, often accompanied by swelling, nausea, and vomiting. In a study conducted in southeast Nigeria involving 34 patients, most patients presented with these classic symptoms, and the authors emphasized that prompt diagnosis and intervention are critical. The average age of patients was 27 years, with many presenting beyond the crucial six-hour window, thereby reducing the chances of testicular salvage [5].

Our case involved a 25-year-old male who presented with left lower abdominal pain and inguinal swelling, symptoms that are atypical for testicular torsion. Initially misdiagnosed as an incarcerated inguinal hernia, the correct diagnosis was considered only after ultrasound findings suggested reduced blood flow to the left testicle. Emergency surgery confirmed and corrected the torsion, preserving the testicle. This atypical presentation underscores the necessity for healthcare providers to maintain a high index of suspicion for testicular torsion in cases of acute lower abdominal pain or inguinal swelling, even in the absence of scrotal pain.

Sathler et al.'s study further illustrates the variability in presentation, describing an intravaginal testicular torsion case in a preschool-aged child, which is less common. They highlighted the unusual age and presentation, adding to the spectrum of atypical cases that healthcare providers must be aware of to avoid diagnostic delays and potential testicular loss [6]. Atypical presentations, such as in our case and the report by Sathler et al., like lower abdominal pain, inguinal swelling, can lead to diagnostic and treatment delays, risking testicular loss. The study from southeast Nigeria highlighted similar challenges, noting that many patients presented late due to intermittent symptoms or atypical presentations. This underscores the need for a thorough clinical evaluation, including a testicular exam, in any male patient presenting with acute abdominal or inguinal pain. Early and accurate diagnosis is vital. Doppler ultrasound is the preferred diagnostic tool, typically showing absent or decreased blood flow to the affected testicle. However, the clinical presentation should guide immediate surgical intervention, as ultrasound findings alone may not always be conclusive.

## Conclusions

Testicular torsion can present atypically, making early and accurate diagnosis both challenging and essential for testicular preservation. Our report highlights the necessity for healthcare providers to broaden their clinical suspicion for torsion across various presentations. When considered alongside findings from previous studies in southeast Nigeria and those of Sathler et al., this report underscores the importance of maintaining vigilance for both typical and atypical forms of testicular torsion to optimize patient outcomes and prevent irreversible testicular loss.
